# Impact of atlas-CT-based bone anatomy compensation on MR-based attenuation correction for brain PET imaging in a time-of-flight PET/MRI system: A direct comparison to a patient-CT-based approach

**DOI:** 10.1186/2197-7364-2-S1-A68

**Published:** 2015-05-18

**Authors:** Jaewon Yang, Yiqiang Jian, Michel Tohme, Spencer Behr, Daniel Vigneron, Sharmila Majumdar, Youngho Seo

**Affiliations:** University of California, San Francisco, USA; GE Healthcare, USA

An atlas-CT-based bone-anatomy compensation for MR-based attenuation correction (MRAC) in brain PET/MRI imaging is a current standard. However, the impact of an anatomical difference has not been clinically evaluated. Thus, we aim to evaluate the impact of the anatomical dissimilarity on MRAC. Whole-body FDG-PET/CT followed by PET/MRI were performed for twelve patients in an integrated TOF PET/MRI system. The MRAC utilized an atlas-CT (MRAC-atlas) as well as a patient-specific-CT (MRAC patient) to produce AC maps (pseudo-CT). Instead of using atlas-CT, the MRAC-patient approach derived pseudo-CT from patient-specific-CT aligned to MR. For quantitative evaluation, CTAC was considered as gold standard for AC, and PET mean activity concentration values were measured and compared in eight 10 ml volumes-of-interest (VOI). PET activity concentration with MRAC, compared to CTAC, were systematically underestimated on average by 0.63±0.34 kBq/ml (4.0±2.2%) and 0.22±0.21 kBq/ml (1.4±1.5%) for the MRAC-atlas and the MRAC-patient, respectively: using the MRAC atlas, the error was increased to 0.41±0.25 kBq/ml (2.6±1.8%) on average (p≈0). However, the error increase was patient-dependent (highest: 5.7% vs. lowest: 0.3%) and VOI dependent (highest 3.1% vs. lowest: 1.9%). For the first time, the atlas-CT based MRAC was compared to the patient-specific-CT-based MRAC for brain PET imaging in an integrated TOF PET/MRI system. Overall, the MRAC-atlas achieves quantification accuracy similar to CTAC with a small but measurable difference of 5% in values, which is 2.6% higher than the error of the MRAC-patient.

